# Role of Apelin in Type II Diabetes Mellitus

**DOI:** 10.7759/cureus.107781

**Published:** 2026-04-27

**Authors:** Mahima Mehra, Sandeep Garg, Praveen Bharti, Ravada R Hemanth Sai Sri Harsha

**Affiliations:** 1 Medicine, Maulana Azad Medical College, New Delhi, IND; 2 Medicine, Maulana Azad Medical College and Associated Lok Nayak Hospital, New Delhi, IND; 3 General Medicine, Maulana Azad Medical College, New Delhi, IND

**Keywords:** apelin, diabetes, diabetic complications, insulin resistance, microvascular and macrovascular complications

## Abstract

Introduction: Diabetes mellitus (DM) is characterised by persistent hyperglycaemia. It is associated with severe microvascular and macrovascular complications and leads to a significant decline in quality of life. Apelin is an endogenous ligand for the APJ receptor. There is some evidence in the literature that it promotes glucose uptake and improves insulin sensitivity and thus helps to control diabetes and prevent its complications in the initial stages. In later stages, however, apelin is found to aggravate the complications of diabetes by causing endothelial dysfunction and microangiopathic changes.

Objective: The aim of our study was to assess apelin levels in patients with type II DM. Our primary objective was to find an association between serum apelin levels and insulin resistance. Secondary objectives were to find an association between serum apelin levels and the control and duration of diabetes, and also the micro- and macrovascular complications of type II DM.

Materials and methods: The study was conducted among the Indian population in view of the massive prevalence of type II DM in India. A total of 60 patients were selected after applying the inclusion and exclusion criteria. A detailed history was taken for the duration and complications of diabetes, and samples were drawn for fasting and postprandial blood sugar levels, HbA1c, LFT (liver function test) and KFT (kidney function test), lipid profile, serum fasting insulin levels, and serum apelin levels. Urine albumin assessment by dipstick and UACR (urine albumin-creatinine ratio) and fundus examination were also done.

Conclusion: The study found significantly high levels of serum apelin among the subjects. It also found significant associations between serum apelin levels and HOMA-IR (homeostatic model assessment for insulin resistance) and fasting blood sugar levels. Apelin levels were also found to be higher in patients with macro- and microvascular complications.

## Introduction

Diabetes mellitus (DM) refers to a group of metabolic disorders characterised by persistent hyperglycaemia [1]. DM is one of the fastest-growing global health conditions, with projections indicating that 693 million adults will be affected by 2045. The disease is associated with severe macrovascular complications, such as cardiovascular disease, stroke, and peripheral vascular disease, as well as microvascular complications, including diabetic nephropathy, diabetic retinopathy, and neuropathy. These complications contribute to heightened mortality rates, blindness, kidney failure, and a significant decline in the overall quality of life for individuals living with diabetes [2]. Apelin, an endogenous ligand for the APJ receptor, a G protein-coupled receptor, is widely distributed within the human body and animals. It plays an important role in various physiological and pathophysiological situations [3]. Apelin plays a key role in regulating glucose metabolism, and the apelin-APJ system is closely associated with DM and its complications. Apelin also promotes glucose uptake, enhances insulin sensitivity, and regulates lipolysis and fatty acid oxidation. Moreover, apelin administration has been found to improve conditions related to diabetes. Consequently, the apelin-APJ system can potentially be a therapeutic target for the treatment of diabetes and its complications [4].

Atherosclerosis, which leads to the narrowing of arterial walls, is the key pathological mechanism in the development of the macrovascular complications. Hyperglycaemia and reactive oxygen species cause inflammatory changes and injury to the arterial wall in the peripheral and coronary vascular systems and are implicated in the pathogenesis of atherosclerosis [5]. Apelin inhibits lipid accumulation and inflammatory factor release in macrophages, thereby preventing atherosclerosis by suppressing lipoprotein lipase in macrophage foam cells. Some studies have also found that apelin may promote atherosclerosis by upregulating NOX4 expression and increasing reactive oxygen species [6].

Diabetic retinopathy is a microvascular complication of diabetes. Early changes in non-proliferative diabetic retinopathy are associated with pericyte loss. Hyperglycaemia-induced loss of pericytes results in increased vascular permeability and damage to endothelial cells, leading to enhanced leakage, immune cell infiltration, and the progression of proliferative diabetic retinopathy. In early stages of diabetic retinopathy, apelin has been found to reduce pericyte apoptosis, enhance endothelial cell-to-cell junctions and create non-leaky blood vessels [7]. In the late stages of proliferative diabetic retinopathy, however, apelin promotes neovascularisation [8].

Another microvascular complication of DM is peripheral neuropathy. A study by Hosny SS et al. found elevated apelin levels in patients with diabetic neuropathy compared with patients with diabetes without neuropathy (p < 0.001), suggesting that elevated apelin levels may indicate endothelial dysfunction and microangiopathic changes in diabetic patients [9]. Apelin improves insulin sensitivity and increases glucose uptake, thus preventing further deterioration of DPN. The microvascular complication of diabetes leading to maximum morbidity and mortality is diabetic nephropathy. A study by Gao Z et al. has shown that apelin plays a protective role against diabetic nephropathy by decreasing insulin resistance and reducing proteinuria [10]. Other studies, however, have shown that apelin promotes the progression of diabetic nephropathy [11].

However, there is a scarcity of studies directly assessing the relationship of serum apelin levels with insulin resistance and diabetes complications in the Indian population. Hence, this study was undertaken to study the role of apelin in DM and its association with insulin resistance and diabetic complications, particularly in the Indian population.

## Materials and methods

This is a cross-sectional observational study conducted by the Department of Medicine, Maulana Azad Medical College and Lok Nayak Hospital, from 1st August 2024 to 31st July 2025. After taking approval from the Institutional Ethics Committee on 03/04/2024 (ID no. F.1/IEC/MAMC/MD/MS(108/01/2024/No.132)), a total of 60 individuals of either sex, aged more than 45 years, and diagnosed with type II DM were included in the study. Patients with other conditions that activate the apelinergic system, such as chronic liver disease, kidney disease due to causes other than diabetes, malignancy, heart failure, and obesity, were excluded from the study.

Patients visiting the Medicine or Endocrinology OPD during the study period were enrolled in the study. All patients were informed about the study design, and informed consent was taken. At the time of enrolment, a detailed history was taken pertaining to the age at onset of diabetes, duration of symptoms, symptoms at onset, and complications of diabetes. Clinical examination was done in each case. Each patient underwent a monofilament test for the detection of neuropathy and a fundus examination for retinopathy. A patient was considered to have diabetic peripheral neuropathy if they were unable to perceive the 10 g monofilament at ≥1 testing site on the foot, with the standard testing sites being the plantar surface of the great toe; 1st, 3rd, and 5th metatarsal heads, heel, and lateral foot.

Biochemical investigations were carried out for parameters such as fasting and postprandial glucose, liver and kidney function tests, urine albumin-to-creatinine ratio (UACR), lipid profile, serum fasting insulin, and HbA1c. A UACR value of >30 mg/g of creatinine was considered indicative of diabetic nephropathy.

A 250 µL sample was collected from each patient and stored at -80 °C for serum apelin levels for up to 90 days. Serum apelin levels were estimated by using a commercially available ELISA kit - the Human APLN (Apelin) ELISA kit (catalogue no. ELK4069; ELK Biotechnology Co., Ltd., Wuhan, China).

The competitive inhibition enzyme immunoassay technique was used in the assay. The microtiter plate was pre-coated with human apelin. Standards and patient samples were added to the wells, followed by a biotin-labelled antibody specific to apelin. Then, avidin linked to horseradish peroxidase (HRP) was added and incubated. After incubation, a substrate solution (TMB) was added, which produced a colour reaction. The reaction was stopped by adding sulphuric acid. The colour intensity was measured using a spectrophotometer at 450 nm. The concentration of apelin in the samples was calculated by comparing their optical density (OD) with a standard curve. The sensitivity of the kit was 8.4 pg/mL [12].

The kit did not specify a standard cut-off for serum apelin levels; however, according to the literature, normal serum apelin levels are 0.8-1.3 ng/mL [13].

Patients underwent a comprehensive evaluation to assess their control and the presence of chronic complications at the time of enrolment. Insulin resistance was assessed by calculating the Homeostatic Model Assessment for Insulin Resistance (HOMA-IR). HOMA-IR was determined using fasting plasma glucose and fasting serum insulin values according to the following formula: HOMA-IR = [Fasting Insulin (µIU/mL) × Fasting Glucose (mg/dL)] / 405. Higher HOMA-IR values more than 2.5 indicate greater insulin resistance.

Statistical analysis

Data were entered into Microsoft Excel (Microsoft Corporation, Redmond, Washington) and analysed using Epi Info (Centers for Disease Control and Prevention, Atlanta, GA, USA), JASP (JASP Team, Amsterdam, Netherlands), and IBM SPSS Statistics for Windows, Version 23 (Released 2015; IBM Corp., Armonk, New York). Continuous variables were expressed as mean ± standard deviation (SD) or median with interquartile range (IQR), while categorical variables were presented as frequencies and percentages. Normality of data distribution was assessed using the Kolmogorov-Smirnov test and graphical methods. Differences between groups were analysed using the chi-square test or Fisher’s exact test for categorical variables and Student’s t-test/ANOVA or Mann-Whitney U test/Kruskal-Wallis test for continuous variables as appropriate. A p-value of <0.05 was considered statistically significant.

## Results

The demographic profile comparison shows participants with a mean age of 55.45 ± 9.21 years (median 52 years, range 45-75 years). Most participants were in the 45-55-year age group (55.0%), and 56.7% were female. The mean duration of DM was 7.85 ± 6.04 years, with the majority of patients (45.0%) having diabetes for 6-10 years. Table 1 shows the demographic profile of participants.

**Table 1:** Demographic profile of the study participants DM: diabetes mellitus; IQR: interquartile range

Clinical Details	Mean ± SD	Median (IQR)	Min–Max OR N (%)
Age (Years)	55.45 ± 9.21	52.00 (47.75–62.00)	45.00–75.00
Age Group			
45­–55 Years	33 (55.0%)	-	-
56–65 Years	16 (26.7%)	-	-
66–75 Years	11 (18.3%)	-	-
Gender			
Male	26 (43.3%)	-	-
Female	34 (56.7%)	-	-
Duration of DM (Years)	7.85 ± 6.04	8.50 (3.00–10.00)	0.50–30.00
Duration of DM			
Newly Diagnosed	6 (10.0%)	-	-
<5 Years	16 (26.7%)	-	-
6–10 Years	27 (45.0%)	-	-
>10 Years	11 (18.3%)	-	-

The apelin levels among the 60 patients with DM enrolled in our study were in the range of 1.52-4.81 ng/mL. The mean apelin levels among our subjects were 3.18 ng/mL. The mean apelin levels in ng/mL among males were 3.15 and among females were 3.20, which were significantly higher than normal serum apelin levels (0.8 to 1.3 ng/mL) in the healthy adult population.

Table 2 shows that serum apelin levels were significantly positively correlated with fasting blood glucose (ρ = 0.29, p = 0.0271), fasting insulin (r = 0.42, p < 0.0015), and HOMA-IR (ρ = 0.51, p < 0.0011). However, the correlations with HbA1c (ρ = −0.05, p = 0.091) and postprandial blood glucose (ρ = 0.21, p = 0.1011) were not statistically significant.

**Table 2:** Correlation of various variables of diabetes with apelin levels Spearman's correlation was used to determine the significance. FBS: fasting blood sugar; PPBS: postprandial blood sugar; HOMA-IR: homeostatic model assessment for insulin resistance

Parameters	Apelin (ng/mL)	p-value
HbA1c (%)	Correlation Coefficient (rho) = -0.05	0.09
FBS (mg/dL)	Correlation Coefficient (rho) = 0.29	0.027
PPBS (mg/dL)	Correlation Coefficient (rho) = 0.21	0.101
Fasting Insulin (µU/mL)	Correlation Coefficient (r) = 0.42	<0.001
HOMA-IR	Correlation Coefficient (rho) = 0.51	<0.001

**Figure 1:**
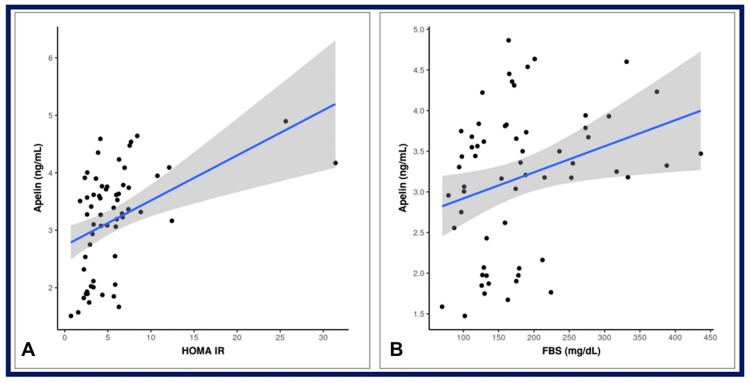
Scatterplot showing correlation of apelin with (A) HOMA-IR and (B) FBS HOMA-IR: homeostatic model assessment for insulin resistance; FBS: fasting blood sugar

Figure 1 shows a scatterplot showing the correlation between serum apelin levels vs. HOMA-IR and fasting blood sugar.

 Table 3 shows apelin levels in patients with and without diabetes complications.

**Table 3:** Apelin levels in patients with and without vascular complications The significance of the correlation between diabetic nephropathy and apelin levels was determined by a t-test. The significance of the correlation of diabetic neuropathy, retinopathy, coronary artery disease, and cerebrovascular accident with serum apelin levels was determined by the Wilcoxon-Mann-Whitney U test. The significance of the correlation between the number of microvascular complications and serum apelin levels was determined by the Kruskal-Wallis test.

Parameters	Apelin (ng/mL)	Test	p-value
Microvascular Complications: Nephropathy		T: 0.637	0.527
Present	3.27 ± 0.93	-	-
Absent	3.12 ± 0.87	-	-
Microvascular Complications: Retinopathy		W: 669	0.001
Present	3.56 ± 0.83	-	-
Absent	2.83 ± 0.80	-	-
Microvascular Complications: Neuropathy		W: 607	<0.001
Present	3.49 ± 0.74	-	-
Absent	2.41 ± 0.78	-	-
Macrovascular Complications: Coronary Artery Disease		W:135	0.495
Present	3.45 ± 1.21	-	-
Absent	3.16 ± 0.87	-	-
Macrovascular Complications: Cerebrovascular Accident		W: 296	0.172
Present	3.61 ± 0.42	-	-
Absent	3.10 ± 0.93	-	-
Macrovascular Complications: Peripheral Vascular Disease		-	-
Present	-	-	-
Absent	3.18 ± 0.89	-	-
Any Microvascular Complications		W: 437	0.006
Present	3.35 ± 0.85	-	-
Absent	2.52 ± 0.77	-	-
Any Macrovascular Complications		W: 402	0.035
Present	3.70 ± 0.49	-	-
Absent	3.05 ± 0.92	-	-
Number of Microvascular Complications		X^2 ^:15.1	0.002
None	2.52 ± 0.77	-	-
1	2.76 ± 0.77	-	-
2	3.55 ± 0.71	-	-
3	3.53 ± 0.90	-	-
Number of Macrovascular Complications		W: 172	0.035
None	3.05 ± 0.92	-	-
1	3.70 ± 0.49	-	-

Microvascular complications of DM, such as retinopathy, neuropathy, and nephropathy, were assessed in this study. Similarly, macrovascular complications assessed were coronary artery disease, cerebrovascular accident, and peripheral vascular disease. Serum apelin levels were significantly elevated in patients with diabetic retinopathy (3.56 ± 0.83 ng/mL vs. 2.83 ± 0.80 ng/mL, p = 0.0014) and diabetic neuropathy (3.49 ± 0.74 ng/mL vs. 2.41 ± 0.78 ng/mL, p < 0.0014), whereas no significant difference was observed in patients with nephropathy or any individual macrovascular complications, such as CAD, CVA, and PVD. Overall, participants with any microvascular complication had higher apelin levels compared to those without (3.35 ± 0.85 vs. 2.52 ± 0.77 ng/mL, p = 0.0064), and those with any macrovascular complication also showed elevated levels (3.70 ± 0.49 vs. 3.05 ± 0.92 ng/mL, p = 0.0354). Furthermore, apelin levels progressively increased with the number of microvascular complications, from 2.52 ± 0.77 ng/mL in patients with none to 3.53-3.55 ng/mL in patients with two or three complications (p = 0.0022), highlighting a strong association between circulating apelin and the burden of diabetic microvascular complications.

**Figure 2:**
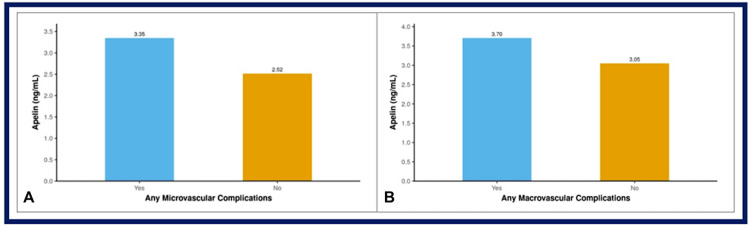
Bar graphs showing comparison of serum apelin levels in patients with and without (A) micro- and (B) macrovascular complications, respectively

The bar graphs in Figure 2 illustrate that serum apelin levels are higher in patients with complications. Specifically, those with any microvascular complications have an average apelin level of 3.35 ng/mL compared to 2.52 ng/mL in patients without such complications. Similarly, patients with any macrovascular complications show higher apelin levels (3.70 ng/mL) than those without (3.05 ng/mL). However, the levels were not significantly associated with any individual macrovascular complication.

**Figure 3:**
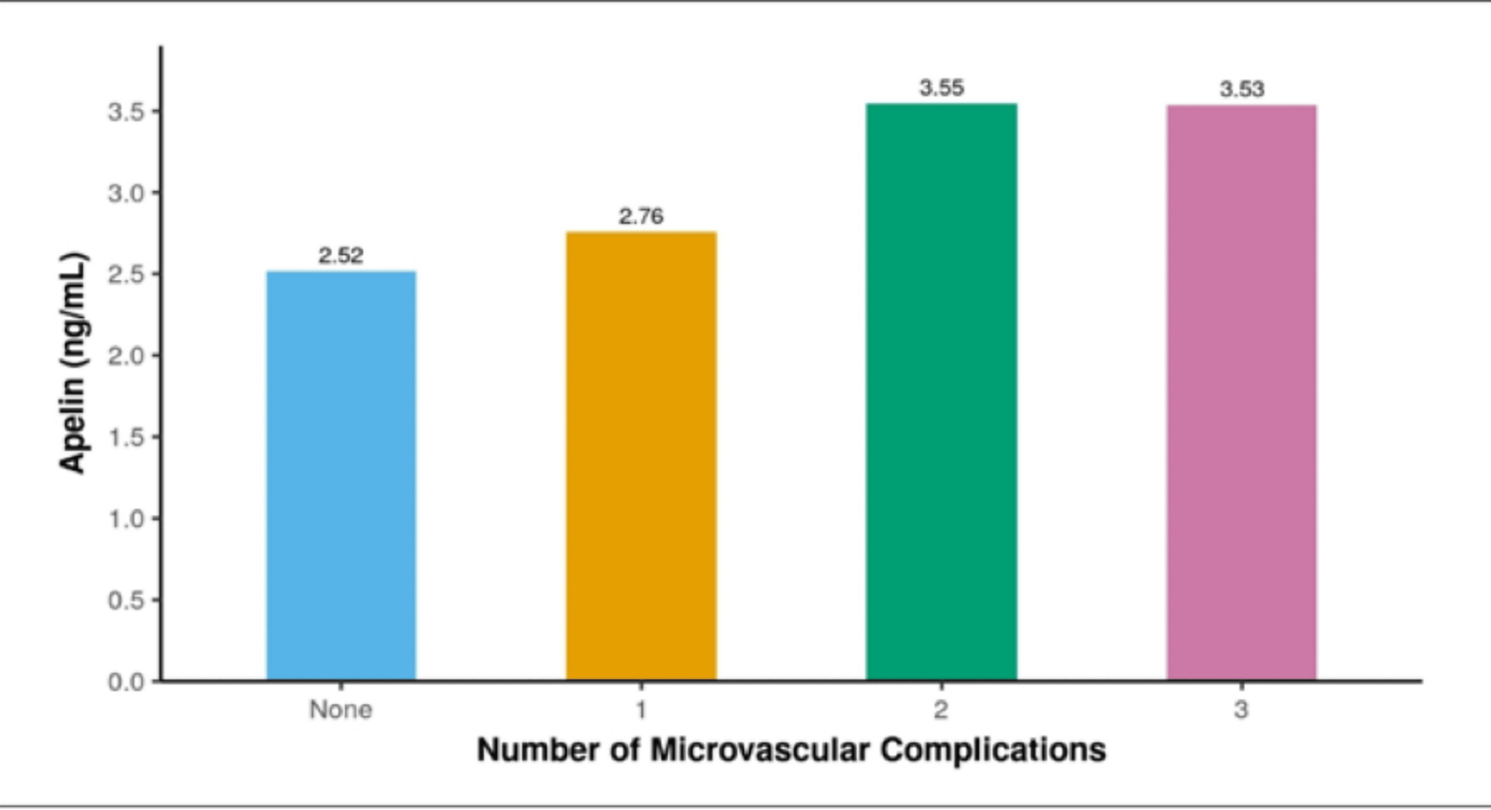
Bar graph showing that patients with more microvascular complications had higher serum apelin levels

The bar graph in Figure 3 shows that serum apelin levels increase with the number of microvascular complications. Patients without any microvascular complications had the lowest apelin level (2.52 ng/mL), while those with two or three complications had significantly higher levels (3.55 ng/mL and 3.53 ng/mL, respectively), indicating a positive association between apelin concentration and the severity of microvascular disease.

Apelin levels were found to be associated with HOMA-IR, fasting blood sugar, the presence of microvascular complications (diabetic retinopathy and neuropathy), and the number of microvascular complications.

## Discussion

DM is a metabolic disorder characterised by hyperglycaemia. Impaired glucose metabolism resulting from insulin resistance causes an increase in serum apelin levels. Apelin is found to cause lipolysis and fatty acid oxidation, promote glucose utilisation, and improve insulin sensitivity. In our study, there was a significant association between insulin resistance and serum apelin levels. HOMA-IR levels positively correlated with serum apelin levels with a correlation coefficient of 0.51(r), suggesting that increased insulin concentration as a result of insulin resistance leads to an increase in apelin secretion by adipocytes.

Our study also found a positive correlation between serum apelin levels and fasting blood sugar levels (ρ = 0.29, p = 0.0271). A similar correlation (r=0.12, p=0.027) between serum apelin levels and fasting blood sugar levels was also found in a study by Cavallo MG et al., as hyperglycaemia leads to hyperinsulinemia, which leads to apelin gene expression in adipocytes, resulting in a compensatory increase in serum apelin levels, which in turn improves insulin sensitivity and glycaemic control [14]. Hyperglycemia also has a direct effect on adipocytes, resulting in an increase in serum apelin levels.

Our study also showed an association between serum apelin levels and the presence (p = 0.006) and number (p = 0.002) of microvascular complications of diabetes. Patients with microvascular complications tend to have higher serum apelin levels, and the levels tend to increase with the number of microvascular complications. The explanation is that microvascular complications are characterised by endothelial injury as a result of chronic hyperglycemia, advanced glycation end products, and oxidative stress, and endothelial cells express the APJ receptor-apelin system. When the endothelium is injured, apelin expression increases; it promotes NO release and improves microvascular perfusion. Thus, higher apelin reflects a compensatory endothelial protective response [15]. A study by Glassford et al. showed that hypoxia results in increased activation of hypoxia-inducible factor-1α, which in turn induces the release of apelin. Apelin responds to hypoxia by inducing angiogenesis as a compensatory response [16]. This suggests that apelin played a role in the pathogenesis and progression of the microvascular complications. Apelin was found to be associated with diabetic neuropathy and diabetic retinopathy. Studies have shown that apelin has a mitotic effect on endothelial cells and promotes angiogenesis. Apelin has been found to play a key role in retinal neovascularisation. Apelin aggravates diabetic neuropathy by causing endothelial dysfunction and microangiopathic changes by causing activation of inflammatory signalling. Apelin binding to the APJ receptor activates intracellular pathways, including NF-κB and MAPK, leading to increased production of TNF-α, IL-1β, and IL-6. These cytokines damage Schwann cells and impair axon regeneration, thus amplifying the injury in peripheral nerves [17].

Our study also showed that, overall, patients with macrovascular complications had higher apelin levels, indicating that apelin was associated with atherosclerosis, the key mechanism behind the development of macrovascular complications. However, we did not find any significant association of apelin levels with any individual macrovascular complication.

The limitations of this study were a small sample size (n=60), which limits statistical power; a single-centre design, which limits generalisability; and its cross-sectional nature, which precludes causal inference. Additionally, our study found no association between serum apelin levels and duration of diabetes. It also found no association between serum apelin levels and HbA1c. A larger sample size, multi-centrality of the study design, and a longitudinal study design could have provided greater insight into trends and would have had greater generalisability. Another limitation is that potential confounders (e.g., BMI, medications, lifestyle factors) were not analysed; however, obesity, chronic liver disease, heart failure and malignancies are the main confounders that independently affect apelin levels, and patients with those comorbidities were excluded from our study.

## Conclusions

This study explored the role of serum apelin in type II DM. We investigated serum apelin levels in patients with diabetes and the associations of apelin levels with insulin resistance, diabetes control, diabetes duration, and micro- and macrovascular complications of diabetes. Key findings included higher apelin levels in individuals with diabetes, a significant association between serum apelin and HOMA-IR and fasting blood sugar, and higher serum apelin levels in patients with a higher number of microvascular complications.

This suggests that serum apelin can serve as a biomarker of insulin resistance and reflects glycaemic control, correlating with fasting blood sugar, suggesting its potential utility as a biomarker for monitoring disease control. Beyond metabolic regulation, apelin is involved in endothelial function, vasodilation, and angiogenesis, implicating it in the development of both microvascular complications and macrovascular complications. Altered apelin levels may thus serve as an early indicator of vascular dysfunction and disease progression. Studying the association of apelin with insulin resistance, glycaemic control, and complications is particularly relevant in populations with a high burden of diabetes, as it may contribute to improved risk stratification and the development of population-specific reference values.
